# Evaluation of antibody responses to outer membrane vesicles (OMVs) and killed whole cell of *Vibrio cholerae* O1 El Tor in immunized mice

**Published:** 2019-06

**Authors:** Manijeh Sedaghat, Seyed Davar Siadat, Esmat Mirabzadeh, Malihe Keramati, Farzam Vaziri, Morvarid Shafiei, Fereshteh Shahcheraghi

**Affiliations:** 1 Department of Microbiology, Pasteur Institute, Tehran, Iran; 2 Department of Mycobacteriology and Pulmonary Research, Pasteur Institute, Tehran, Iran; 3 Department of Biotechnology Research, Pasteur Institute, Tehran, Iran; 4 Department of Pilot of Nano-Biotechnology, Pasteur Institute, Tehran, Iran

**Keywords:** Outer membrane vesicle, *Vibrio cholerae* O1 El Tor, Killed whole cell, Dukoral vaccine

## Abstract

**Background and Objectives:**

Cholera disease remains an important global health problem affecting 3–5 million subjects worldwide. Outer membrane vesicles (OMVs) have been found in a variety of Gram-negative bacteria and act as protective transport vesicles. The aim of this study was to evaluate Immune responses against *Vibrio cholerae* O1 El Tor clinical strain OMV and compare it with killed whole cell (KWC), complex of (KWC-OMV) as well as the internationally licensed oral cholera vaccine, Dukoral, in serum and intestinal secretions of mice.

**Materials and Methods:**

OMVs were prepared by using modified detergent-centrifugation procedure from *V. cholerae* O1 El Tor clinical strain from 2005 outbreak. The ultrastructure and content of OMVs were investigated via the Scanning Electron Microscopy (SEM) and SDS-PAGE analysis. Three doses of oral immunization were adjusted and total IgG and IgA in serum and intestinal secretion were measured by enzyme-linked immunosorbent assay (ELISA).

**Results:**

Extracted OMVs from the *V. cholerae* were spherical vesicles with a size ranging from 10 to 300 nm. OMV-immunized mice showed an increased level of total IgG and IgA both in serum and intestinal secretion when compared to the negative controls. Also, there existed a higher level of secretory IgA than the total IgG, suggesting the most of protection against *V. cholerae* colonization provided by sIgA.

**Conclusion:**

Our findings revealed that oral immunization with *V. cholerae* OMVs might induce a long-term immunity, especially when administered in combination with KWC. This study tested the adjuvant activity of OMVs and may be useful in future nano vaccine research.

## INTRODUCTION

As an important Gram-negative bacterium, *Vibrio cholerae* is responsible for a severe diarrheal disease, cholera, which is endemic in Asia and Africa where it can affect five million cases each year. Despite the effective fluid rehydration therapies, cholera remains as a serious global health problem causing approximately 120,000 annual deaths (1). Cholera has always been endemic in most provinces of Iran (2). The bacterium could be transmitted via the fecal-oral route and through contaminated food or water (3), requiring an infectious dose of 10^3^ to 10^11^ organisms which depends on various factors such as blood type, age, health condition and diet type of the patients. If untreated, patients experience the fluid loss, resulting in hypotensive shock, acidosis, and subsequently death after few hours of diarrhea. Although oral and intravenous rehydration therapy have been shown to be effective, there are limitations such as the lack of adequate availability in rural areas and particularly during the outbreaks, the failure of medical facilities to fully support patients with acute diarrhea (4, 5).

Since 2010, WHO has recommended the use of oral cholera vaccines for administration in highly endemic areas and during cholera epidemics. There are two licensed vaccines are available, both of which are killed *V. cholerae* O1 whole cell vaccines. Dukoral vaccine consists of a mixture of Ogawa and Inaba serotypes, El Tor and classical biotypes containing cholera toxin B–subunit (CTB). Shanchol vaccine lacks CTB but contains a *V. cholerae* strain of the O139 serogroup. The latter caused large epidemics of cholera in Bangladesh and India during 1992–1993 (6). Although both types of oral vaccines are immunogenic, they confer a relatively short-term protection.

Dukoral is the only WHO-prequalified oral cholera vaccine. This vaccine requires cold storage, trained health care staff, and is formulated in a suspension in the presence of buffer; resulting in large package volumes and expensive transport chains. The expensiveness of Dukoral has limited its usage in developed countries. Moreover, no vaccine is licensed for under 2 children and none of the commercially available vaccines offer protection against *V. cholera* O139 (7). Therefore, novel cholera vaccines that can be efficiently be administered, distributed, stored and available to all patients around the world is urgently needed.

Outer membrane vesicles (OMVs)-based vaccines were developed more than 20 years ago against *Neisseria meningitidis* serogroup B. OMVs are composed of blebs produced naturally by growing cells and do not arise from cell lysis or cell death. Delivery of toxins, enzymes, and DNA to eukaryotic cells, as well as supporting bacterial survival and pathogenesis are among the functions attributed to OMVs (8). Experimental evidence have shown that the proper use of OMVs protects mice from infections with *N. meningitidis, Salmonella enterica* serovar Typhimurium, *H. pylori*, and *V. cholerae* (9).

In the present study, we focused on oral immunization of BALB/c mice with different vaccine regiments: *V. cholerae* OMV, (KWC-OMV), KWC alone, and Dukoral vaccine, aiming to evaluate the immune responses in sera and intestinal secretion of mice.

## MATERIALS AND METHODS

Bacterial strain used in the study included *V. cholerae* O1 El Tor (ATCC 14033) as a reference strain. The other strain, VC492 (*V. cholerae* O1 El Tor biotype Inaba), was representative of *V. cholerae* and obtained from patients during the 2005 outbreak in Iran. Ribotyping, Pulsed-field gel electrophoresis (PFGE) and PhenePlate (PhP) techniques revealed the clonal dissemination of a single *V. cholerae* strain during that outbreak (10). The strains were stored in 15% glycerol plus brain heart infusion broth (Difco, USA) at −70°C.

### Preparation of OMVs.

As described earlier, vesicles were isolated (11, 12). One litter of Luria broth (LB, Difco, USA) was inoculated with 10 mL of a stationary phase culture of *V. cholera*, followed by growth for 8 h to reach the late exponential-phase at 37°C with aeration. The cell harvest was then inactivated for 30 min at 56°C, cultured on LB agar to confirm the absence of viable bacteria after 24 h, and centrifuged (1 h, 4500×g, 4°C). The cell pellet was resuspended in PBS and centrifuged again (1 h, 4500×g, 4°C). The cell pellet was subsequently resuspended in 0.1 M Tris-10 mM, EDTA buffer, 7.5 times greater than wet weight. Vesicles were extracted by adding 1/20 v/v of 0.1 M Tris, 10 mM EDTA, DOC (Deoxycholate Sodium) (100 g/L) buffer. Vesicles again were separated from cell debris (90 min, 9500×g, 4°C) using Sigma, 3K30 centrifuge, followed by concentrating them through an additional centrifugation (2 h, 60000× g, 4°C). The OMV pellet was resuspended in 0.1 M Tris, 10 mM EDTA, DOC (5 g/L) buffer and the suspension was centrifuged again (2 h, 60000× g, 4°C). Afterwards, the supernatant was passed through 0.45 μm and 0.22 μm pore size filters (PVDF, syringe filters, Germany). Phenyl methyl sulfonyl fluoride (PMSF, Sigma), a protease inhibitor, was added to the filtrated supernatant to prevent protein from degradation. The concentrated OMV was resuspended in 3% sucrose solution and stored at −70°C until use.

### Heat- inactivation of *V. cholerae*.

The bacterial suspension was inactivated by heating in a water-bath at 56°C for 1 h and in order to test the complete killing of bacteria, suspensions were spread on blood agar plates and incubated at 37 °C for 24 h (13).

### Protein assay.

The amount of protein in outer membrane vesicles was measured by Nanodrop (Thermo Scientific, Wilmington, DE, USA) instrument and Bradford assay, followed by using 12% SDS-PAGE (sodium dodecyl sulfate polyacrylamide gel electrophoresis) for the separation of protein molecules based on their molecular weight. After electrophoresis, proteins were stained by 0.1% (w/v) Coomassie blue (14).

### Electron microscopy.

OMVs of bacteria were washed in 0.1 M phosphate buffer, applied to a poly L-lysine coated coverslip, fixed with 2.5% glutaraldehyde, prepared for SEM analysis by sputtering them with a thin gold film (15), and finally imaged via Electron Microscopy (Hitachi S4160, Korea).

### Animal model.

Seven weeks old female BALB/c mice weighting 16–18 g were purchased from the animal resource division of the Pasteur Institute of Iran. The mice were caged separately and kept at 25°C with 50% humidity. They were fed with sterile food and water. All experiments on these mice including immunization and blood collection was approved by the ethical committee of animal care of Pasteur Institute of Iran (Registration No.IR.PII.REC.2015.81). All animals were purchased 1 week before the commencement of experiments, for their adaptation to the environment.

### Oral Immunization.

Mice were divided into 4 groups (n=7 per group) and were immunized orally with 3 doses (days 0, 14 and 28). The immunization doses of regimens were as follows: VC492 (OMV, 25 μg/200 ml PBS), VC492 (KWC) and commercial vaccine (5×10^8^ cell /mL), complex of VC492 (OMV-KWC, Equivalent amounts). Before oral immunization, each mouse was inoculated directly with sodium bicarbonate into the stomach via a mouse feeding needle. The control (non-immunized) groups received PBS. All immunized and non-immunized groups of mice were returned to their cages and received food and water ad libitum within 56 days (8 weeks) post immunization. Non-immunized mice were also housed along with immunized mice during the experiment.

### Collection of serum and intestinal lavage.

Blood samples from immunized and non-immunized control mice were collected from the lateral tail vein on days 0, 7, 14, 21, 28, 35, 42, 49, 56. The collected blood was allowed to clot at room temperature (RT) for 30 min, and centrifuged for 10 min at 4°C and 6000 × g. Serum was isolated and stored at −20°C. Intestinal lavage was collected 35 days after the first immunization and mice (n=3 in each group) were euthanized with an overdose of anesthetic substances, followed by rapid dissection of spinal cord. Afterward, the abdomen was opened and the small intestine was removed, the intestinal loop was washed with 2 ml of PBS+ Protease Inhibitor Cocktail (Sigma) and the liquid resulted from intestinal wash was collected, with adding PMSF and vortexing vigorously. In the next step this mixture was centrifuged at 10,000 × g for 10 min to remove intestinal debris. The supernatant was stored at −20°C for further use (16).

### ELISA.

To determine antibodies titers, enzyme-linked immunosorbent assays (ELISAs) were performed in a procedure described in previous works (17). Disposable polystyrene microtiter wells (Merck, Germany) were separately coated with 100 μL of live *V. cholerae* from reference strain 14033 serovar O1, biotype El Tor, serotype Inaba (10^9^ cells/mL); and incubated overnight at 4°C. Wells were washed three times with PBS (pH 7.4) and blocked with 200 μl of 5% bovine serum albumin (BSA, Merck) for 2 h at 37°C. Wells were washed three times with PBS-T (PBS with 0.5% Tween-20, Sigma) and incubated with diluted serum samples collected from each mouse at 37°C for 1 h. 100 μl HRP-conjugated goat anti-mouse immunoglobulin total IgG (1:100000) and also IgA (1:25000) (Abcam, UK) were added to the wells. Plate was incubated at 37°C for 1 h. After washing with PBS, 3, 3′, 5, 5′-tetramethylbenzidine (TMB) substrate was added to each well, followed by incubation for 15 min in a dark place. The reaction was stopped by adding 100 μl of 2 N sulphuric acid and the plate was read at 450 nm wavelength using an ELISA plate reader (Biotek ELX 800). These experiments were performed in triplicate for each immunoglobulin and on both the immunized and non-immunized serum.

### Statistics.

Prism software system Graph Pad 6 and Student’s t-test was used for all statistical analyses in serum and fecal samples of immunized and control groups. P values of <0.05 were considered as statistically significant.

## RESULTS

### *V. cholerae* OMVs.

**Fig. 1.**
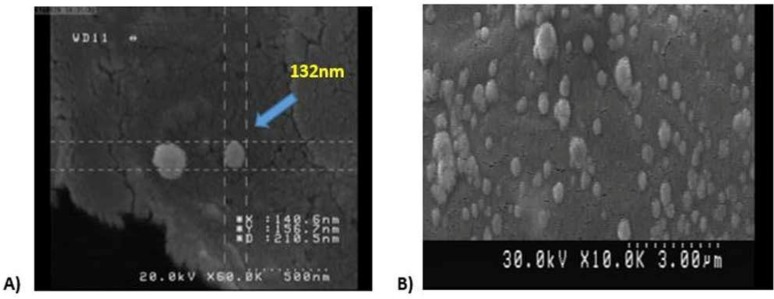
(A, B) Scanning electron micrograph of VC492 outer membrane vesicles (Bar = 50–300 nm), OMV size is shown in panel (A)

**Fig. 2.**
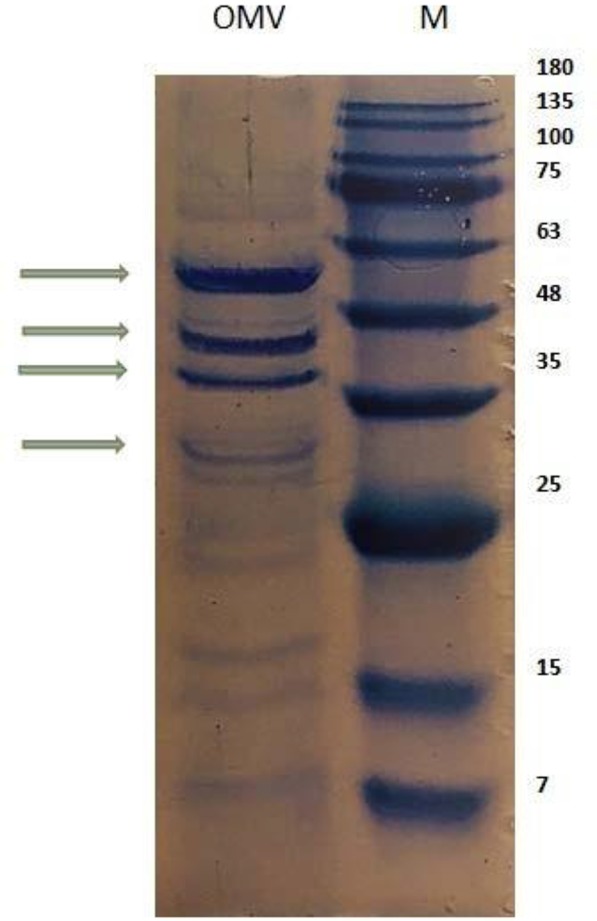
VC492 analyzed OMVs by gel electrophoresis, SDS-PAGE followed by a R250 Blue Coomassie stain. OMVs of VC492 (left line, shown by arrows) and the molecular weight marker in kDa (right line).

In the present study, the immunogenic performance of OMV in a clinical *V. cholerae* strain (VC492) was evaluated. Extraction method with minor modifications was considered a useful tool for OMVs derived VC492, which increased the yield of vesicles with spherical nanostructures to 70–80% with a scale of 100–300 nm (Fig. 1). The total amount of outer membrane vesicles protein was 1.28 and 1.32 mg/mL. In OMV electrophoretic pattern assessed by 12% SDS-PAGE, bands similar to other studies were found, including 33, 37.46.60. kDa bands (Fig. 2).

### Serum antibody responses after immunization.

**Fig. 3.**
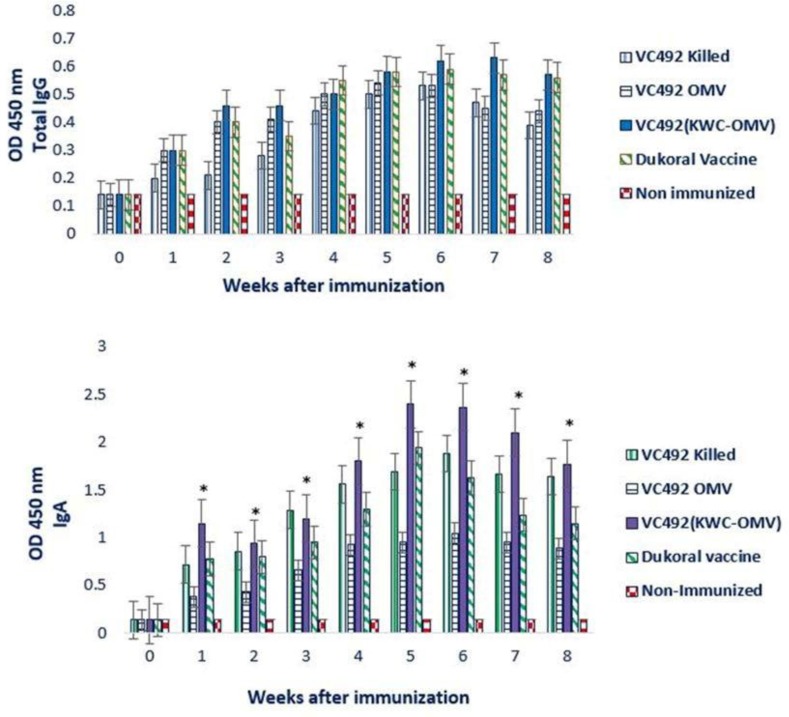
Total IgG and IgA titers in immunized and non-immunized mice sera within 56 days. Four vaccine regimens increased antibody titers (total IgG and IgA) in sera, indicating significant antibody responses in treatment groups in comparison with negative controls after each immunization,) P value < 0.05). IgA levels against VC492 (KWC-OMV) regimen showed significant differences compared to current vaccine, (*P value < 0.05).

In our study, we immunized BALB/c mice with oral administration of three regimens as described in materials and methods section. Serum antibody titers were monitored at nine-time points before, during, and after the Immunization period within 56 days. IgA levels peaked at weeks 5, 6 and 7 post immunization for all immunized groups, while the peak level of total IgG was observed at weeks 5–6. Both types of antibodies gradually decreased after week 7 (Fig. 3).

### Secretory antibody responses.

Our findings showed that the antibody production increased in the fecal pellets of immunized mice after 35 days. Titers of total IgG immunoglobulin in immunized and non-immunize mice indicated significant immune responses compared with control groups, substantial difference were also found in titers of secretory IgA between immunized and non-immunized mice after 35 days of immunization.

**Fig. 4.**
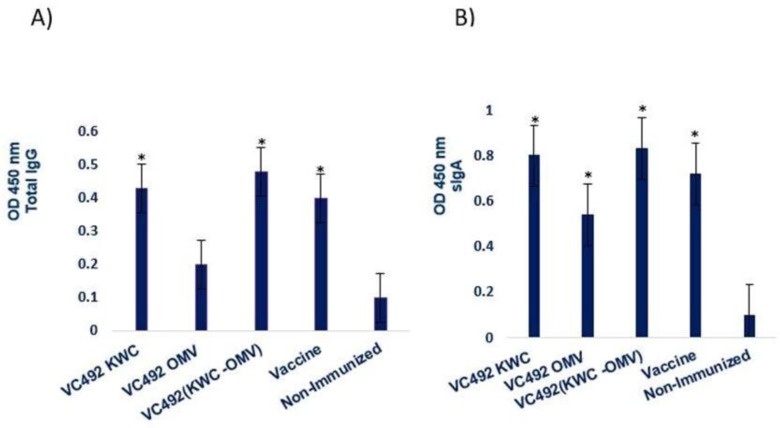
Titers of total IgG (A) and sIgA (B)immunoglobulin in immunized and non-Immunized mice after 35 days. In Panel A: all immunized groups, except those received OMV, showed significant antibody levels compared to control groups, *P value < 0.05, and in panel B: Significantly higher antibody levels were found in immunized groups than the control groups, *P value < 0.05.

This research showed more significant increase in IgA and total IgG levels through immunization with VC492 (KWC-OMV) in the fecal pellets of immunized mice, when compare to the other regimens (Fig. 4).

## DISCUSSION

The global morbidity and mortality of cholera is difficult to be determined, which is due to failure in reporting. However, the annual worldwide prevalence may reach nearly 3–5 million cases (18). A novel generation of vaccines against cholera is highly required to ward off the disease.

OMVs act as protective transport vesicles for delivering toxins, enzymes, and DNA to eukaryotic cells (19). Several lines of evidence regarding the meningococcal OMVs have revealed that these vesicles naturally take advantage of several important features required for a good vaccine, since they contain the composition of the outer membrane (OM) and therefore express some of the most immunogenic and important antigens. Consequently, they can be used as high efficient tools (20). OMVs are stable even at the room temperature and require neither cold chain nor buffer solution, thus making them cost-effective especially for developing countries (21). Studies have shown that purified recombinant CTB (rCTB) protein from *V. cholerae* in combination with inactivated *V. cholera*e whole cells from Iranian strains enhances antibody responses and protection in rabbit animal model (22).

We used a predominant strain, *V. cholerae* O1 El Tor Inaba, which was collected from patients through the 2005 outbreak in Iran (10), aiming to evaluate humoral and mucosal immunity after immunization of female mice with VC492-derived OMVs.

In present study, oral route immunization was used, since it is proved to be an appropriate strategy to eliminate the risk of possible infections transmitted by contaminated needles and syringes (HIV, HepB. HepC). Furthermore, it is not painful and also there is no need for nurses to perform immunization. It has been demonstrated that using chemical detergents such as deoxy cholate sodium for extraction of OMVs not only reduced toxicity of lipopolysaccharide (LPS) but also increased the amount of OMVs (23). In this study, a detergent-centrifugation procedure was used as an appropriate method for extraction of OMVs of VC492. The size of OMVs was fit perfectly with *V. cholerae* OMVs used in other studies (8, 24), which were numerous spherical vesicles with varying sizes, ranging from 100 to 300 nm in diameter.

In our study, levels of serum antibodies (Total IgG, IgA) were high during the first day of inoculation and gradually decreased until day 56, indicating significant immune responses in comparison with control groups. Also, a higher serum level of IgA was found in comparison with total IgG. Moreover, a substantial IgA antibody responses was found in sera by administration (KWC-OMV) of clinical strain regimen, compared to commercial vaccine. We also evaluated secretory IgA (sIgA) and IgG total titers in the fecal pellets of immunized mice with four vaccine regimens after 35 days. Studies have shown that IgA might hamper the attachment and intestinal colonization of *V. cholerae* (25).

In present study, immunized mice showed a higher level of sIgA than total IgG in the fecal pellets, suggesting that sIgA is likely to provide most of protection against *V. cholerae* colonization at the mucosal surface of gastrointestinal tract.

Overall, oral immunization of female mice with three doses of OMVs of *V. cholerae* induced remarkable antibody responses in serum and intestinal lavage and long-term immunity especially in VC492 (KWC-OMV) regimen; making it more immunogenic and protective than OMV, KWC alone, and commercial vaccine. This immunization also induced stronger humoral responses and led to the significant production of mucosal antibodies in mice, which may be due to antigenic enhancement, increasing the yield of antibody, or linked to the adjuvant properties of OMV. Although our findings are promising, other challenges need to be taken into consideration. It appears to be useful to investigate the correlation between serum bactericidal assay (SBA) and ELISA for measuring antibody responses against *V. cholerae.* In conclusion, the present work demonstrated key features of VC492 predominant strain and revealed humoral and mucosal immunogenicity against this strain of *V. cholerae*. It is worth mentioning that VC492 (KWC-OMV) induced long term protection in mice and it can be considered in the vaccine studies in the future on the other hand adjuvanticity of OMV and increasing knowledge on OMVs over the past few years pave the way for the development of the next generation of novel vaccine formulations in the form of Nano-Vaccine.
